# Detection and Characterization of a Novel Picornavirus in European Badger (*Meles meles*)

**DOI:** 10.3390/vetsci9110645

**Published:** 2022-11-21

**Authors:** Andrea Palombieri, Paola Fruci, Vittorio Sarchese, Serena Robetto, Riccardo Orusa, Alessio Arbuatti, Vito Martella, Barbara Di Martino, Federica Di Profio

**Affiliations:** 1 Department of Veterinary Medicine, Università degli Studi di Teramo, 64100 Teramo, Italy; 2 Centro di Referenza Nazionale per le Malattie degli Animali Selvatici (CeRMAS), Istituto Zooprofilattico Sperimentale del Piemonte, della Liguria e della Valle d’Aosta, 11020 Aosta, Italy; 3 Department of Veterinary Medicine, Università Aldo Moro di Bari, 70010 Valenzano, Italy

**Keywords:** *Picornaviridae*, sakobuvirus, fecal virome, badger

## Abstract

**Simple Summary:**

A molecular survey was performed to investigate the gut virome of wild mustelids and sciurids found dead in Northwestern Italy. Using pan-picornavirus primer pair, we discovered a new picornavirus (PV) in the intestinal content of a European badger (*Meles meles*). The full-length genome of this novel strain was obtained by a sequence-independent single-primer amplification procedure in combination with Oxford Nanopore Technologies sequencing platform. On sequence analysis, the badger PV could be considered the prototype of a new species, proposed as Sakobuvirus B, classified within the still poorly characterized genus *Sakobuvirus*. The finding of this study poses interesting questions about the genetic diversity of these viruses, suggesting that the PV host range could be wider than expected.

**Abstract:**

The recent development of unbiased metagenomic next-generation sequencing has provided a richer view of the wild animal virome making it necessary to expand the knowledge about virus diversity in wildlife, as well as to monitor their potential transmission to domestic animals or humans. In the present study, by screening collections of enteric specimens from wild animals, a novel picornavirus was identified in the intestinal content of a badger (*Meles meles*). By enrichment with a sequence-independent single-primer amplification (SISPA) approach and deep sequencing with Oxford Nanopore Technologies (ONT) platform, the genome sequence of a novel picornavirus strain, Badger/3A-2019/ITA, was reconstructed. On comparison based on the polyprotein sequences, the virus was distantly related (58.7% and 59.7% sequence identity at the nucleotide and amino acid level, respectively) to the feline picornavirus strain FFUP1, identified in 2012 in Portugal and classified into genus *Sakobovirus* within the species *Sakobuvirus A*. Upon phylogenetic, pairwise homology, and distance analyses performed on the P1, 2C^hel^, 3C^pro^, and 3D^pol^ proteins and the complete genomic sequence, the badger picornavirus may be considered a member of a new sakobuvirus species, which we propose as Sakobuvirus B.

## 1. Introduction

The family *Picornaviridae* includes important human and animal pathogens, associated with a wide range of diseases and, in some cases, with zoonotic potential. Picornaviruses (PVs) are small, icosahedral, non-enveloped viruses, with highly diverse positive-sense single-stranded RNA genomes with lengths ranging from 6.7 to 10.1 kb. The viral genome is predicted to encode a single polyprotein that is processed by viral proteinases to produce the structural proteins 1A, 1B, 1C, and 1D, also referred to as VP4, VP2, VP3, and VP1, and the non-structural proteins (NSPs) 2A^pro^, 2B, 2C^hel^, and 3A, 3B^VPg^, 3C^pro^, 3D^pol^ [1]. Advances in diagnostics and deep sequencing technologies have dramatically increased the number of newly identified PVs in humans and animals, revealing the ubiquitous nature and a surprising degree of diversity of genome sequence and layouts. By broad-range consensus reverse transcription (RT)-PCRs and unbiased analysis of nucleic acids from fecal samples, novel PVs have occasionally been identified also in domestic carnivores (dog and cat) and in wildlife, including ferret, fox, golden jackal, side-striped jackal, spotted hyena, and wolf [2,3,4,5,6,7,8,9,10,11,12,13,14]. This has raised questions, in particular on their epidemiology in animal hosts, their clinical impact, as well as their zoonotic potential and spill over infections [15]. Herein, we report the identification and genetic characterization of a novel PV detected in the fecal sample collected from a badger during a molecular survey aimed to investigate the gut virome composition in the carcasses of different species of wild mustelids and sciurids.

## 2. Materials and Methods

### 2.1. Sampling

The collection included a total of 38 enteric samples obtained from 10 stone martens (*Martes foina*), 17 European badgers (*Meles meles*), 2 otters (*Lutra lutra*), 6 marmots (*Marmota marmota*), and 3 squirrels (*Sciurus vulgaris*). Sampling was carried out in the framework of a National passive surveillance program, by the National Reference Centre for Wild Animal Diseases (CeRMAS—IZS PLV) in different regions of north-western Italy (Liguria, Piemonte and Valle d’Aosta Regions) between February and September 2019. 

### 2.2. Molecular Investigation for PVs

Fecal samples were subjected to RNA extraction using TRIzol LS (Invitrogen, Ltd., Paisley, UK) and screened by broadly reactive consensus reverse transcription (RT)-PCR assay for PVs [3]. RT-PCR was performed using SuperScriptTM One-Step RT-PCR (Invitrogen, Ltd., Paisley, UK). Amplicons of expected size, analysed by 1.5% agarose gel electrophoresis, were purified using a QIAquick gel extraction kit (Qiagen GmbH, Hilden, Germany) and subjected to direct sequencing using BigDye Terminator Cycle chemistry and 3730 DNA Analyzer (Applied Biosystems, Foster, CA, USA). Basic Local Alignment Search Tool (BLAST; http://www.ncbi.nlm.nih.gov, accessed on 1 September 2022) and FASTA (http://www.ebi.ac.uk/fasta33, accessed on 1 September 2022) with default values were used to find homologous hits. 

### 2.3. Oxford Nanopore Technologies (ONT) Sequencing

In order to acquire the complete viral genome sequence of a badger strain detected by a pan-picornavirus degenerate RT-PCR protocol [3] (Table 1) and also to investigate the virome composition of the positive fecal sample, a sequence-independent single-primer amplification (SISPA) protocol [16] was performed, using primers FR26RV-N [17], FR40RV-T [18] and FR20RV [17] (Table 1). The PCR-enriched sample was quantified by Qubit dsDNA HS assay (Invitrogen, Ltd., Paisley, UK) and used for library preparation and for adapter-ligation with Genomic DNA by Ligation kit SQK-LSK110 (Oxford Nanopore Technologies, ONT, Oxford, UK). A clean-up step was carried out using AMPure XP beads (Beckman Coulter, Brea, CA, USA). The library was loaded onto flow cell Flongle FLO-FLG001 (vR9.4.1) and sequenced on a MinION Mk1C (ONT, Oxford, UK) device for 8 h. ONT MinKNOW software (v3.1.5), was used to collect raw sequencing data and ONT cloud-based basecaller based on GUPPY (v3.2.8) was employed to perform on-site and real-time basecalling during the sequencing run. Genome Detective Virus Tool Premium (https://www.genomedetective.com/db/ui/login, accessed on 1 September 2022) [19] was employed to perform sequence trimming, filtering, and assembling of viral reads. A total of 1,930,717 viral reads (average length of 900 bases) generated from the badger sample was mapped and assembled to a PV of feline origin, strain FFUP1 (GenBank accession no. KF387721), currently classified within the species *Sakobuvirus A* in the genus *Sakobuvirus* [8]. The resulting draft consensus sequence was subjected to BLASTn search. The alignment of the sequences was carried out using MAFFT multiple alignment program [20] v. 7.388 plugin of the Geneious Prime v. 2022.2.2 (Biomatters Ltd., Auckland, New Zealand). Phylogenetic analysis was conducted using Maximum likelihood method based on the Poisson correction and supplying statistical support with bootstrapping of 1000 replicates, in MEGA X software (v1.0) [21].

### 2.4. Virus Isolation

Attempts were made to cultivate the sakobuvirus in BHK-21 (Baby Hamster Kidney 21) and Vero (African green monkey kidney) cells lines. Briefly, the badger fecal sample was filtered with 0.22 μm filters, inoculated into 90% confluent cells in 24-well plates and incubated at 37 °C in a 5% CO_2_ incubator. After an adsorption period of 45 min, DMEM was added. Viral growth was evaluated daily monitoring the onset of cellular cytopathic effect (CPE) for 5 days. The cryolysates were sub-cultured three times into fresh monolayers and collected for RNA extraction. The presence of sakobuvirus RNA was verified using RT-PCR as described above.

### 2.5. RT-PCR for Sakobuviruses

In order to investigate if the novel sakobuvirus circulates actively in wild animals, all fecal samples collected from wild mustelids and sciurids were re-screened using a pan- sakobuvirus RT-PCR assay based on broadly reactive primers (Table 1) designed by visual inspection of an alignment containing the genome sequences of the badger strain, the feline sakobuvirus A (SaKoV-A) [8], the wild boar strain (unpublished data), and the corresponding conserved regions of sequences identified in fur seals [22]. 

## 3. Results

Out of 38 fecal samples collected from mustelids and sciurids, virological screening revealed positivity only in a badger specimen (2.6%, 1/38) when tested using pan-picornaviruses primer pair targeting a fragment of 125 bp of the 3D^RdRp^ encoding region [3]. Upon direct sequencing and preliminary analyses with BLAST and FASTA, the badger sequence displayed the highest nucleotide [nt] identities to the feline SaKoV-A strain FFUP1 (KF387721) (63.8%) [8], to a complete genome sequence (62.9%) identified in a wild boar fecal sample available on GenBank database (MW660837) and also to an unclassified sakobuvirus sequence (KR072982) (43.6%) detected in a fur seal found deceased along the coast of the state of Rio Grande do Sul (Brazil) [22].

The complete genome of the strain SaKoV/Badger/3A-2019/ITA (GenBank under accession no. OP293080) was reconstructed using ONT. Out of 6,638,705 filtered reads obtained, 2,598,992 were classified as viral. Of these, 1,930,717 sequences were mapped to the feline SaKoV-A strain FFUP1 generating a single contig of 7813-nt in length with a depth of coverage of 1284489.5. The genome of the strain 3A-2019/ITA showed the same organization 5′-UTR-[L-1AB-1C-1D-2A^H-Box/NC^-2B-2C/3A-3B-3C-3D]-3′-UTR as the feline SaKoV-A strain FFUP1. The complete 3′-UTR was 169 nt long, whilst a 372 nt-long portion of the 5′-UTR was determined, shorter than the 5′-UTR region (591-nt) of SaKoV-A. Downstream the 5′-UTR, a single ORF of 7272 nt in length, encoding a large polyprotein of 2423 amino acids (aa), was mapped. The putative translation initiation codon (AUG) of the strain SaKoV/Badger/3A-2019/ITA (corresponding to position 592–594 of feline SaKoV-A), was predicted at nt position 373–375, located in a weak Kozak consensus (GNNAUGC) [23]. Whilst most of the protease-cleavage sites were shared with SaKoV-A, distinct aa residues were found at the junctions of L and 1AB (Q/G), 1C and 2A (Q/A), and 3B and 3C (Q/S). Similar to the feline SaKoV-A, the badger sakobuvirus contained conserved aa motifs of PVs, including the H-box/NC motif of the 2A protein, the nucleotide-binding site GxxGxGKS (_1401_GAPGVGKS_1408_) and helicase motif DDxxQ (_1452_DDIGQ_1456_) of the 2C, and the _2115_KDE_2117_, _2245_PSG_2247_, _2283_YGDD_2286_ and _2332_FLKR_2335_ motifs of the 3D protein. In the complete coding region, the strain SaKoV/Badger/3A-2019/ITA shared the highest identity to feline SaKoV-A (58.7% nt and 59.7% aa) and to the wild boar sakobuvirus strain WBSA (54.3% nt and 56.2% aa identities). Genetic similarities were also found to PVs of the genus *Kobuvirus* (49.8% nt and 43.0% aa identities), *Salivirus* (46.8% nt and 38.5% aa identities), and *Passerivirus* (45.5% nt and 37.1% aa identities). 

**Figure 1:**
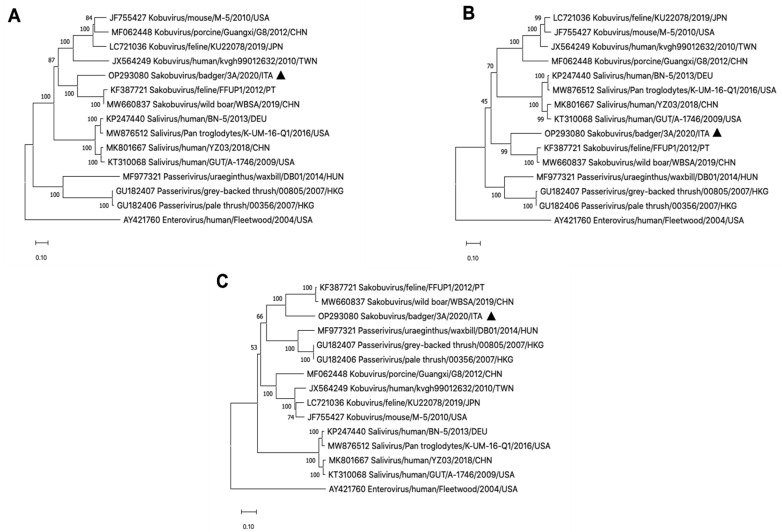
Phylogenetic analyses based on the complete amino acid sequence of the coding regions of P1 (**A**), 2C^hel^ (**B**), 3C^pro^/3D^pol^ (**C**) of the strain SaKoV/Badger/3A-2019/ITA (GenBank accession no. OP293080). Phylogenetic trees were constructed by using Maximum likelihood method based on the Poisson correction and supplying statistical support with bootstrapping of 1000 replicates. The black triangle indicates the badger strain detected in this study. Evolutionary analyses were conducted in MEGA X [21].

One of the criteria established by the ICTV *Picornaviridae* Study Group (http://www.picornastudygroup.com/defnitions/genus_defnition.htm, accessed on 10 September 2022) for establishing a new picornavirus genus is a significant divergence of the orthologous protein sequences, exceeding 66% for P1 (precursor of capsid proteins 1AB, 1C, 1D) and 64% for NSPs 2C^hel^, 3C^pro^ and 3D^pol^. Conversely, no criteria are currently available for picornavirus species demarcation within the genus *Sakobuvirus*, although strains of different species are expected to display a significant divergence of P1, 2C^hel^, 3C^pro^, and 3D^pol^ proteins and to form distinct monophyletic groups in the phylogenetic tree [15]. In our analysis, pairwise sequences comparison between the badger strain and the feline SaKoV-A revealed aa identities of 61.5% in the P1 polyprotein, and of 65.0%, 57.5%, and 69.9% in the NSPs 2C^hel^, 3C^pro^, and 3D^pol^, respectively, supporting the classification of the badger PV within the genus *Sakobuvirus*. On the other hand, maximum likelihood-based phylogenetic trees constructed on the P1, 2C^hel^, 3C^pro^/3D^pol^ proteins yielded similar topologies (Figure 1), with strain SaKoV/Badger/3A-2019/ITA segregating along with the genome sequences of feline and wild boar sakobuviruses, currently classified in the species *Sakobuvirus A*, although in a well-distinct branch. Accordingly, the badger virus could be considered the prototype of a novel species, for which we propose the name Sakobuvirus B. 

Although attempts to isolate the virus, in both cell cultures inoculated signs of CPE after all the serial passages were not observed and the RT-PCR reactions showed no visible products in the expected size in any of the cryolysates. Furthermore, all fecal samples, re-screened using the consensus RT-PCR based on the pan-SaKoV primer, tested negative. 

## 4. Discussion

Information on the epidemiology of sakobuviruses is still limited. The first identification of these novel PVs was documented in Portugal in 2012 on the metagenomic analysis of a fecal sample collected from a clinically healthy cat [8]. The virus named SaKoV-A was classified in a novel picornavirus genus designated *Sakobuvirus* within the species *Sakobuvirus A.* Subsequent molecular screening using highly specific primers revealed the presence of a viral sequence genetically closely related to the prototype strain (99.0% nt identity) in an additional fecal sample collected from a diarrhoeic cat, with an overall prevalence of 3.6% (2/55) [8]. With exception of this study, there are no other reports of SaKoV-A infection in cats. More recently, the full genome of a sakobuvirus has been found in a wild boar fecal sample (MW660837, unpublished data), showing 86.4% nt and 90.9% aa identities to the feline SaKoV-A. In addition to *Sakobuvirus A*, a new putative species within the genus *Sakobuvirus* was identified in 2013 during a metagenomic study aimed to investigate the fecal virome composition of two species of fur seals found deceased along the shore of Rio Grande do Sul (Brazil) [22]. All the contigs generated displayed the highest aa identity (ranging from 58.0% to 73.0%) to the feline SaKoV-A, with the reads covering 59% of the complete polyprotein. The identification of a potential new sakobuvirus species in the intestinal content of a badger extends data on the genetic heterogeneity of PVs, also suggesting an unexpected variety of ecological niches. Since we analysed the feces, we could not determine if the detected virus was able to infect and replicate actively in badger or it was rather transported passively through the gastrointestinal tract, thus reflecting a dietary and/or environmental contamination. Testing other tissues/organs of badgers and large-scale surveillance studies could help establish the potential impact, if any, of sakobuviruses on badger health and its possible role as an enteric pathogen. 

## 5. Conclusions

A growing number of novel PVs have been recently identified in wild and domestic animals in association with enteric disease. However, for many of them, epidemiological surveys are still limited, making difficult to achieve any firm conclusions on their enteropathogenic role [2,3,24,25,26,27]. In addition, a plethora of PVs has been discovered serendipitously as components of the enteric virome in clinically healthy animals [4,7,9,12,28,29]. Whether these orphan viruses have the ability to cause disease is still uncertain, so further studies are awaited before viral pathology can be ascertained.

Finally, gathering data on the virome of wild animals is pivotal to depict a baseline of virus diversity in wildlife and assess the possible threat to animal welfare and conservation and, eventually, to assess possible risks for public health.

## Figures and Tables

**Table 1:** List of primers used in this study. Nucleotide position refers to feline SaKoV-A strain FFUP1 (GenBank accession no. KF387721).

Primers	Target Genes	Assay	Sequence (5′ to 3′)	Position	References
3D-for	3D^RdRp^	Screening RT-PCR	GTGGGCTGCAAYCCNGA	7438–7454	[3]
3D-rev	3D^RdRp^	Screening RT-PCR	TTNAGNGCATCAAACCARA	7544–7563	[3]
FR26RV-N	--	cDNA synthesis	GCCGGAGCTCTGCAGATATC-N6	--	[17]
FR40RV-T	Poly-(A) tail	cDNA synthesis	GCCGGAGCTCTGCAGATATC-T20	--	[18]
FR20RV	---	SISPA	GCCGGAGCTCTGCAGATATC	--	[17]
SaKoV-for	3D^RdRp^	pan-SaKoV RT-PCR	GGTAGCGCGGTCGGTTGCGACCC	6862–6884	This study
SaKoV-rev	3D^RdRp^	pan-SaKoV RT-PCR	CCCAGGACTGGTAGTTGTTAG	7529–7550	This study

## Data Availability

The data that support the findings of this study are available from the corresponding author upon reasonable request.
